# Severe obesity in a specialist type 2 diabetes outpatient clinic: an Australian retrospective cohort study

**DOI:** 10.1186/s12902-021-00722-9

**Published:** 2021-03-24

**Authors:** Arunav Thakur, Dharmesh Sharma, Bhavya Gupta, Nikitha Kramadhari, Rohit Rajagopal, David Simmons, Milan Kumar Piya

**Affiliations:** 1 School of Medicine, Western Sydney University, Campbelltown, New South Wales 2560 Australia; 2 Macarthur Diabetes Service, Camden and Campbelltown Hospitals, Campbelltown, New South Wales 2560 Australia

**Keywords:** Obesity, Health care delivery, Type 2 diabetes, Bariatric

## Abstract

**Background:**

Obesity is a major risk factor for the development of type 2 diabetes (T2DM) and its complications. Significant weight loss has been shown to improve glycaemia in people with T2DM and obesity. National and international guidelines recommend considering bariatric surgery for body mass index (BMI) ≥ 35 kg/m^2^. We assessed the proportion of people with T2DM meeting criteria for surgery, how many had been offered a bariatric/obesity service referral, and compared the characteristics of people with BMI ≥ 35 kg/m^2^ and BMI < 35 kg/m^2^.

**Methods:**

Retrospective data were collected for all people with T2DM aged ≥18 years, attending a hospital specialist diabetes outpatient service over three calendar years, 2017–2019.

**Results:**

Of 700 people seen in the service, 291 (42%) had BMI ≥ 35 kg/m^2^ (the “BMI ≥ 35 group”) and met criteria for bariatric surgery, but only 54 (19%) of them were offered referral to an obesity service. The BMI ≥ 35 group was younger than those with a BMI < 35 kg/m^2^ (56.1 ± 14.8 vs 61.4 ± 14.6 years, *p* < 0.001) (mean ± SD), with similar diabetes duration (11.0 ± 9.0 vs 12.3 ± 8.9 years, *p* = 0.078), and there was no significant difference in initial HbA1c (75 ± 27 vs 72 ± 26 mmol/mol, *p* = 0.118) (9.0 ± 2.5 vs 8.7 ± 2.4%) or proportion treated with insulin (62% vs 58%). There was more GLP1 agonist use in the BMI ≥ 35 group (13% vs 7%, *p* = 0.003) but similar rates of SGLT2 inhibitor use (25% vs 21%, *p* = 0.202). The BMI ≥ 35 group received more new medication and/or dose adjustments (74% vs 66%, *p* = 0.016). Only 29% in the BMI ≥ 35 kg group achieved HbA1c < 53 mmol/mol (7.0%).

**Conclusions:**

In spite of frequently meeting the criteria for bariatric surgery and not achieving glycaemic targets, people with T2DM in this specialist clinic received limited medical or surgical management of their obesity. This study suggests opportunities for improvement in care of people with T2DM at several levels including increased referrals from T2DM services to weight management/bariatric services, as well as an increased use of GLP1 agonists and SGLT2 inhibitors where appropriate. Our data support the need to prioritise obesity management in the treatment of type 2 diabetes.

## Background

Type 2 diabetes mellitus (T2DM) is a growing problem with a worldwide prevalence of approximately 7% in adults, and a similar prevalence across Australia [1, 2]. Obesity is associated with an increase in mortality with increasing body mass index (BMI), and is a well-established risk factor for type 2 diabetes [3]. Obesity (BMI ≥ 30 kg/m^2^) prevalence worldwide is 13% [4], and 11% of adults in Australia are living with severe obesity (BMI ≥ 35 kg/m^2^) [5]. Beyond the major health challenge, the additional annual total health cost, in comparison to those with a normal weight without diabetes, is 26% for obesity alone and 46% for people with obesity and diabetes [6].

Glycaemic control is important in the context of T2DM, particularly in reducing the risk of microvascular complications [7]. In addition, the 10-year follow-up data from United Kingdom Prospective Diabetes Study showed a continued reduction in microvascular and macrovascular risk for participants who underwent intensive glucose control, suggesting a benefit from early good control, the legacy effect [8]. However, in people with longstanding T2DM, particularly those with existing cardiovascular disease or risk factors, the benefits of pushing glycaemic targets lower to reduce cardiovascular disease and mortality are less clear [9]. Weight loss, if significant and sustained, can improve glycaemic control as shown in the DiRECT trial, DIADEM-1 trial and Look AHEAD study [10–12]. However, sustained weight loss is difficult to achieve in clinical practice, whether it be with lifestyle or pharmacotherapy. In spite of this, weight and diabetes outcomes have been shown to be significantly better for people with obesity and T2DM in a multidisciplinary obesity service when compared to a hospital diabetes clinic [13]. Bariatric surgery is also an established treatment option for severe obesity and T2DM, and can result in significant, sustained weight loss and improvement/remission of T2DM [14, 15]. Bariatric surgery data and the DiRECT and DIADEM-1 trials have all shown that intervening soon after the diagnosis of T2DM is necessary for diabetes remission with weight loss [10, 11, 16]. Hence, the European Association for the Study of Diabetes (EASD) - American Diabetes Association (ADA) consensus guidelines for T2DM management recommend treating obesity, alongside glycaemia, with weight-lowering medications and bariatric surgery [17].

The bulk of T2DM is managed in primary care with only a small proportion being seen in a specialist diabetes clinic, usually those with diabetes complications. These clinics are limited in numbers and average local waiting times to see new patients are over 6 months from referral date, with similar waiting times in nearby publicly funded clinics and shorter waiting times of 2–3 months in private clinics. Various models of integrated care have been proposed to improve patient care and reduce the burden on specialist clinics [18, 19].

In Australia, the National Health and Medical Research Council (NHMRC) Guidelines recommend considering bariatric surgery for people with T2DM and a BMI ≥ 35 kg/m^2^ [20]. This is corroborated by multiple international guidelines including the UK National Institute for Health and Clinical Excellence (NICE) guidelines and Diabetes Surgery Summit [21, 22]. Between July 2018 and June 2019, less than 10% of primary bariatric surgeries in Australia were publicly funded [23], and there is limited access to publicly funded specialised obesity services as reported in a position statement by the Clinical Obesity Services in Public Hospitals (COSIPH) in Australia [24]. As a result, many people with severe obesity and T2DM may not receive timely referrals to obesity services or have bariatric surgery discussed, as it is not readily available for them. At the same time, specialist diabetes clinics in hospitals may not be able to provide timely optimal management for T2DM or help with weight management. Therefore, in people with T2DM attending a specialist diabetes outpatient clinic, our aims were:

1. To describe the proportion that met the recommended criteria for bariatric surgery using a cut-off of ≥ 35 kg/m^2^.
2. To compare the characteristics and outcomes in people with a BMI ≥ 35 kg/m^2^ to those with a BMI < 35 kg/m^2^.
3. To determine the proportion of people with BMI ≥ 35 kg/m^2^ that were referred to the metabolic clinic or had bariatric surgery discussed with them as an option.
4. To compare metabolic outcomes between people who were new to the clinic, and people who had been attending the clinic in previous years.

## Participants and methods

### Design

Retrospective data were collected for all non-pregnant adults with T2DM attending the specialist diabetes outpatient service in a public teaching hospital in Sydney.

The following inclusion criteria were applied:

- Type 2 diabetes
- Age ≥ 18
- Had attended an appointment with an endocrinologist for type 2 diabetes between 01/01/17 and 31/12/19

**Fig. 1:**
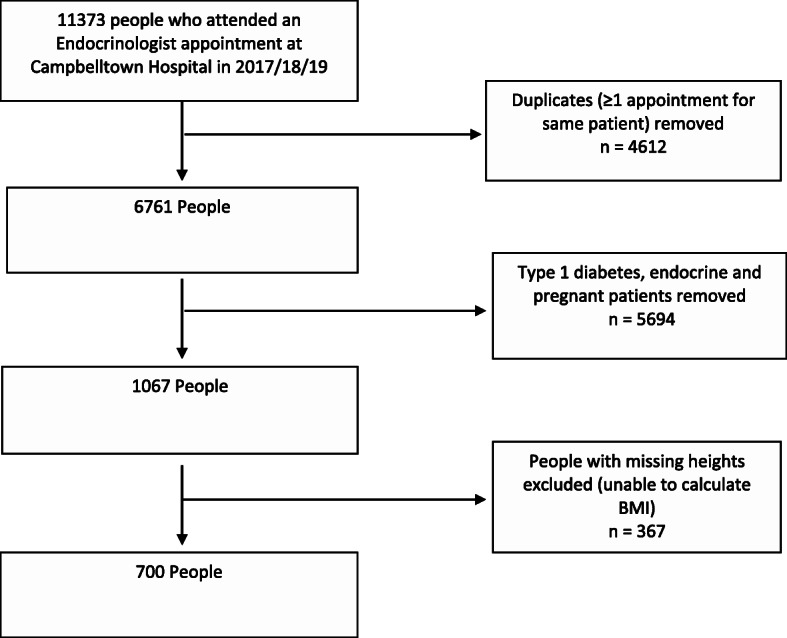
Data Collection Flowchart

A list of all people who saw an endocrinologist across the three calendar years was generated from the hospital’s electronic medical records. The database was then filtered to exclude type 1 diabetes, diabetes in pregnancy, or people with a general endocrine/thyroid condition alone. This database was then cross-checked with patient notes and correspondence letters to select all the people with T2DM. Those with no recorded height were excluded due to the inability to calculate their BMI. The data collection flow chart is shown in Fig. 1.

Patient electronic medical records (eMR) were used to obtain demographic data, pathology results, anthropometric data, use of insulin and other anti-diabetes medications, complications of diabetes, annual screening, patient education and referrals or discussions of bariatric surgery. This was corroborated by checking patient correspondence letters. The study was approved by the South West Sydney Local Health District (SWSLHD) Research Ethics Committee as a quality improvement project (Reference: CT20_2018).

We compared baseline data and follow up metabolic outcomes in people with a BMI ≥ 35 kg/m^2^ to those with a BMI < 35 kg/m^2^. Some people only had one HbA1c reading available in a calendar year, and hence they had to be excluded when calculating HbA1c reduction and proportion reaching HbA1c < 53 mmol/mol (7.0%). Amongst the BMI ≥ 35 kg/m^2^ group, we also compared outcomes in people who were new referrals to the clinic to existing patients who had been attending the clinic in previous years.

A formal sample size calculation was not performed, but the number of patients across 3 years was felt to be adequate for the specified outcomes. Data were analysed using SPSS software, version 26 (SPSS Inc., Chicago, Ill, USA). Distribution of continuous variables was tested for normality using the Kolmogorov–Smirnov test. Means and standard deviations were computed for continuous variables and frequencies and percentages for categorical variables. T-tests were performed for continuous variables and chi-square tests for categorical variables. *P*-value of < 0.05 was considered statistically significant. All tests were 2 tailed.

## Results

Of the 700 eligible people, 42% had a BMI ≥ 35 kg/m^2^. Almost a quarter of people (23%) had a BMI ≥ 40 kg/m^2^. The BMI ≥ 35 kg/m^2^ group was younger, with a similar diabetes duration, and a higher proportion of women (Table 1). Among the BMI ≥ 35 kg/m^2^ people, only 19% (*n* = 54) were either offered referral to an obesity clinic, or had bariatric surgery discussed with them. The BMI ≥ 35 kg/m^2^ group attended a higher number of dietitian appointments on average (0.3 ± 0.7 vs 0.2 ± 0.5, *p* = 0.019) but there was no difference between both groups for endocrinologist (1.8 ± 1.6 vs 1.9 ± 1.8) and diabetes educator (specialist nurse) appointments (0.7 ± 1.3 vs 0.7 ± 1.3) (Table 2). There was no statistical difference in starting HbA1c (75 ± 27 vs 72 ± 26 mmol/mol, *p* = 0.118) (9.0 ± 2.5 vs 8.7 ± 2.4%) and the two groups had a similar reduction in HbA1c between their first and last readings (15 ± 28 vs 14 ± 28 mmol/mol) (− 1.4 ± 2.6 vs − 1.2 ± 2.5%) (Table 2).

**Table 1:** Baseline Data

Measure	BMI ≥35 kg/m^2^ (*n* = 291) 42%	BMI < 35 kg/m^2^ (*n* = 409) 58%	*P* value
Age	56.1 ± 14.8	61.4 ± 14.6	**< 0.001**
%Women	53.3% (*n* = 155)	42.3% (*n* = 173)	**0.004**
Weight (kg)	119.6 ± 24.3	81.9 ± 16.2	**< 0.001**
BMI (kg/m^2^)	42.5 ± 7.0	28.8 ± 3.8	**< 0.001**
Duration of Diabetes (years)	11.0 ± 9.0	12.3 ± 8.9	0.078
%Smoker/Ex-Smoker	31% (*n* = 90)	28% (*n* = 115)	0.421
Hypertension	70% (*n* = 202)	66% (*n* = 268)	0.270
Systolic BP	131 ± 18	130 ± 18	0.265
Diastolic BP	76 ± 11	75 ± 11	0.418
Dyslipidaemia	59% (*n* = 171)	59% (*n* = 241)	0.939
Cholesterol Medication	53% (*n* = 154)	61% (*n* = 247)	**0.047**
Total cholesterol	4.5 ± 1.3	4.2 ± 1.4	0.138
LDL	2.3 ± 1.0	2.2 ± 0.9	0.223
HDL	1.1 ± 0.3	1.2 ± 0.9	0.504
Triglycerides	2.8 ± 1.8	2.7 ± 3.4	0.786
Obstructive Sleep Apnoea	25% (*n* = 73)	6% (*n* = 24)	**< 0.001**
Ischaemic Heart Disease	25% (*n* = 71)	26% (*n* = 104)	0.734
Stroke	9.7% (*n* = 28)	7.9% (*n* = 32)	0.411
Chronic Kidney Disease Stage 3 or below	19% (*n* = 55)	22% (*n* = 91)	0.261
eGFR	70.2 ± 22.8	67.7 ± 23.9	0.217
Abnormal Urine ACR	51% (*n* = 107)	47% (*n* = 116)	0.838
Peripheral Vascular Disease	6.2% (*n* = 18)	5.4% (*n* = 22)	0.660
Peripheral Neuropathy	18% (*n* = 52)	18% (n = 73)	0.987
Current/Previous Foot ulcer	8.6% (*n* = 25)	3.9% (*n* = 16)	**0.009**
Previous lower limb amputations	4.1% (*n* = 12)	2.4% (*n* = 10)	0.210
Laser treatment for Diabetic Retinopathy	4.3% (*n* = 12)	6.4% (*n* = 26)	0.257
Depression	16% (*n* = 46)	11% (*n* = 44)	**0.049**
Starting HbA1c	75 ± 27 mmol/mol (9.0 ± 2.5%)	72 ± 26 mmol/mol (8.7 ± 2.4%)	0.118
% on Insulin	62% (*n* = 180)	58% (*n* = 235)	0.252
Total daily dose of insulin (units)	59.9 ± 49.3	37.2 ± 26.0	**0.004**

*eGFR* Estimated glomerular filtration rate, *ACR* Albumin/creatinine ratio, *LDL* Low density lipoprotein, *HDL* High density lipoprotein, *BP* Blood pressure

**Table 2:** Glycaemic control and clinic attendance

Measure	BMI ≥ 35 kg/m^2^ (*n* = 291) 42%	BMI < 35 kg/m^2^ (*n* = 409) 58%	*P* value
Final HbA1c ^a^	66 ± 20 mmol/mol (8.2 ± 1.8%)	62 ± 19 mmol/mol (7.8 ± 1.7%)	0.073
HbA1c reduction % ^a^	15 ± 28 mmol/mol (1.4 ± 2.6%)	14 ± 28 mmol/mol (1.2 ± 2.5%)	0.661
% achieving HbA1c < 53 mmol/mol (7.0%) ^a^	29%	39%	0.092
% with HbA1c > 75 mmol/mol (9.0%) ^a^	29%	19%	0.066
Documented hypoglycaemia	21% (*n* = 59)	30% (*n* = 121)	**0.007**
Frequent hypoglycaemic episodes	9.5% (*n* = 27)	14% (*n* = 56)	0.084
Documented severe hypoglycaemia	1.4% (*n* = 4)	2.3% (*n* = 9)	0.418
Lipohypertrophy	5.3% (*n* = 15)	2.0% (*n* = 8)	**0.019**
Endocrinologist Appointments Attended/patient	1.8 ± 1.6	1.9 ± 1.8	0.788
Total Diabetes Specialist Nurse (Educator) Appointments Attended/patient	0.7 ± 1.3	0.7 ± 1.3	0.998
Total Dietician Appointments Attended/patient	0.3 ± 0.7	0.2 ± 0.5	**0.019**

^a^ *n* = 153 (BMI ≥ 35) and *n* = 119 (BMI < 35) had 2 HbA1c readings available

**Fig. 2:**
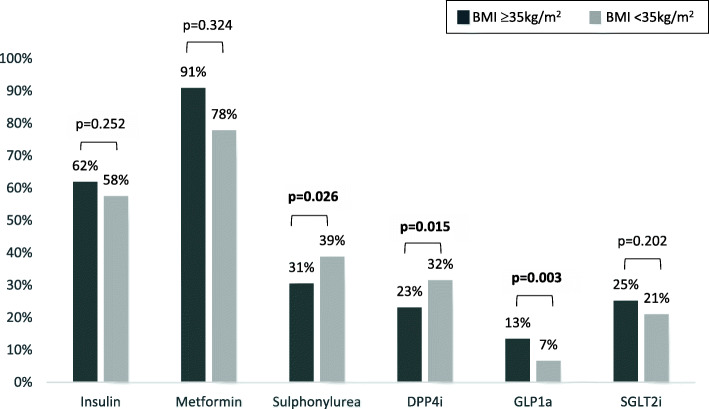
Use of different classes of medications. DPP4i – Dipeptidyl-Peptidase 4, GLP1a – Glucagon-like Peptide 1 agonist, SGLT2i – Sodium/glucose Co-transporter 2 inhibitor

A similar proportion of people were treated with insulin (62% vs 58%) and oral anti-diabetes agents including SGLT2 inhibitors, with fewer patients in the BMI ≥ 35 kg/m^2^ group on sulphonylureas (31% vs 39%) (Fig. 2). In the BMI ≥ 35 kg/m^2^ group, there was more GLP1 receptor agonist use (13% vs 6.6%, *p* = 0.003) but less DPP4i use (23% vs 32%, *p* = 0.015) (Fig. 2). The BMI ≥ 35 kg/m^2^ group had a higher total daily insulin dose (59.9 ± 49.3 vs 37.2 ± 26.0, *p* = 0.004) (Table 1) and a higher rate of lipohypertrophy (5.3% vs 2.0%, *p* = 0.019), but suffered from less documented hypoglycaemia (21% vs 30%, *p* = 0.007) (Table 2). There was also less cholesterol-lowering medication use in the BMI ≥ 35 kg/m^2^ group (53% vs 61%, *p* = 0.047) (Table 1). Obstructive sleep apnoea (25% vs 5.9%, *p* < 0.001), foot ulcers (8.6% vs 3.9%, *p* = 0.009) and depression (16% vs 11%, *p* = 0.049) were more prevalent in the BMI ≥ 35 kg/m^2^ group, with no difference in other co-morbidities including hypertension, dyslipidaemia, ischaemic heart disease, stroke, chronic kidney disease, peripheral vascular disease, peripheral neuropathy, autonomic neuropathy and diabetic retinopathy (Table 1). People with a BMI ≥ 35 kg/m^2^ were more likely to have additional medications/dose increases to diabetes medications during their consultation (74% vs 66%, *p* = 0.016), but there were no differences in annual screening, or patient education regarding hypoglycaemia, driving, and target HbA1c.

Table 3 compares new patients to the clinic and existing clinic patients with BMI ≥ 35 kg/m^2^. New patients had a higher HbA1c, shorter diabetes duration, greater metformin therapy, educator and dietitian attendance, and had a greater HbA1c reduction in clinic than existing clinic patients. Existing patients were more likely to be treated with insulin and/or sulphonylureas, but there was no significant difference in new diabetes agent use (SGLT2 inhibitors and GLP-1 receptor agonists). In the BMI ≥ 35 kg/m^2^ group, HbA1c dropped significantly for people in their first year of attendance, but not for those who had attended the clinic in previous years (− 18 ± 31 vs − 3 ± 16 mmol/mol, *p* < 0.001) (− 1.6 ± 2.8 vs − 0.3 ± 1.4%) (Table 3). However, there was no difference in the proportion reaching HbA1c < 53 mmol/mol (7%) in the first year of clinic attended between those with BMI ≥ 35 kg/m^2^ and BMI < 35 kg/m^2^ groups (31% vs 38%, *p* = 0.261).

**Table 3:** New People (first year in clinic) vs Existing People (from previous years) (BMI ≥ 35 kg/m^2^)

Measure	New (*n* = 208) 50%	Existing (*n* = 207) 50%	*P* value
Age	54.2 ± 14.9	61.0 ± 12.4	**< 0.001**
%Women	51% (n = 107)	56% (n = 116)	0.348
Weight (kg)	120.6 ± 24.8	118.4 ± 21.4	0.334
BMI (kg/m^2^)	42.4 ± 7.2	42.8 ± 6.5	0.570
Duration of Diabetes (years)	10.0 ± 9.0	13.5 ± 8.6	**< 0.001**
Starting HbA1c ^a^	78 ± 27 mmol/mol (9.3 ± 2.5%)	66 ± 21 mmol/mol (8.2 ± 1.9%)	**< 0.001**
HbA1c reduction (%) ^a^	18 ± 31 mmol/mol (1.6 ± 2.8%)	3 ± 16 mmol/mol (0.3 ± 1.4%)	**< 0.001**
% achieving HbA1c < 53 mmol/mol (7.0%) ^a^	31%	23%	0.205
% with HbA1c > 75 mmol/mol (9.0%) ^a^	33%	22%	0.116
% on Insulin	59% (*n* = 123)	70% (*n* = 144)	**0.027**
Metformin	83% (*n* = 173)	75% (*n* = 156)	**0.041**
Sulphonylurea	27% (*n* = 56)	41% (*n* = 85)	**0.003**
DPP4i	25% (*n* = 51)	24% (*n* = 49)	0.842
GLP1 agonist	18% (*n* = 37)	20% (*n* = 41)	0.684
SGLT2i	23% (*n* = 48)	32% (*n* = 65)	0.057
Endocrinologist Appointments Attended/patient	1.8 ± 1.8	1.8 ± 0.9	0.878
Total Diabetes Specialist Nurse (Educator) Appointments Attended/patient	0.8 ± 1.5	0.4 ± 0.9	**< 0.001**
Total Dietician Appointments Attended/patient	0.4 ± 0.7	0.2 ± 0.5	**< 0.001**

*DPP4i* Dipeptidyl peptidase-4 inhibitor, *GLP1 agonist* Glucagon-like peptide-1 receptor agonist, *SGLT2i* Sodium-glucose transport protein 2 inhibitor

^a^*n* = 94 (new) and *n* = 82 (existing) had 2 HbA1c readings available

## Discussion

This study showed that almost half of the people attending the specialist T2DM clinic have a BMI ≥35 kg/m^2^ and meet the criteria for bariatric surgery, and less than a quarter achieve an HbA1c < 53 mmol/mol (7%). However, only 1 in 5 of the people that met the criteria were offered a referral to an obesity/metabolic clinic or for bariatric surgery. This study also showed that the BMI ≥ 35 kg/m^2^ group were younger but already had diabetes for an average of 11 years, had higher insulin requirements and were more likely to have additional medications or doses added to their regimen during consultation. They had a similar diabetes duration to the BMI < 35 kg/m^2^ group, with no difference in initial HbA1c.

The high proportion of people with BMI ≥ 35 kg/m^2^ in our clinic aligns with an audit of a specialist diabetes clinic in the UK, which revealed that 52% of participants with T2DM had obesity (BMI ≥ 30 kg/m^2^) and a further 8.1% had a BMI ≥ 40 kg/m^2^ [25]. The people with obesity were younger, had worse glycaemic control, higher blood pressures, and were more likely to be on an antihypertensive or lipid lowering medication compared to the people with a BMI < 30 kg/m^2^ [25]. Data from the UK’s National Diabetes Audit (NDA) showed little association between BMI and adverse outcomes, apart from an inverse gradient for stroke and MI. Similarly, in our study, the BMI ≥ 35 kg/m^2^ did not have a higher rate of microvascular or macrovascular complications compared to the BMI < 35 kg/m^2^ group [26], although the BMI ≥ 35 kg/m^2^group was younger. As expected, the prevalence of OSA was much higher in the BMI ≥ 35 kg/m^2^ group (26% vs 6.5%, *p* < 0.001) [27]. It is possible that many people in this diabetes clinic have undiagnosed OSA, given that previous studies have demonstrated much higher prevalence of OSA in a specialist diabetes outpatient population [27]. The BMI ≥ 35 kg/m^2^ group also had a higher prevalence of foot ulcers, thus aligning with a previous study which showed a positive correlation between obesity and diabetic foot ulcers [28].

The low proportion of referrals for surgery or obesity/metabolic clinics in our study may be attributed to a combination of lack of publicly funded obesity services and availability of bariatric surgery [23, 24]. However, previous studies have shown that there is also a reluctance in both patients and health care professionals to opt for bariatric surgery due to bias, negative media and attitudes towards weight-loss surgery [29, 30]. Data from our local weight management program has shown that in people with T2DM and a BMI ≥ 40 kg/m^2^ benefitted from improved glycaemic control and reduced diabetic medication load after 6 months of attending the clinic [31], significantly more so than the BMI ≥ 35 kg/m^2^ population in this study. Another retrospective cohort study from Australia, consisting of people with T2DM and BMI ≥ 30 kg/m^2^, compared glycaemic control in participants attending a multidisciplinary weight management clinic to participants receiving “best practice” care in a specialist diabetes clinic [13]. At 30 months, the people attending the weight management clinic achieved a greater HbA1c reduction than those attending the diabetes clinic [13]. This suggests that our BMI ≥ 35 kg/m^2^ population, who had similar baseline characteristics to the participants in the cohort study, are more likely to achieve better glycaemic control by attending a weight management clinic even if bariatric surgery was not available to the patient. This may be because of the multidisciplinary nature of weight management programs that have greater dietitian and psychologist support in weight management programs, with the data here showing very few patients in the clinic were seen by a dietitian or diabetes specialist nurse, while a psychologist was not part of the T2DM service. Therefore, the BMI ≥ 35 kg/m^2^ group may have been better served in the multidisciplinary obesity service, leaving more capacity for the specialist diabetes clinic to see more patients with T2DM and its complications.

In the Look AHEAD trial, people with type 2 diabetes and overweight/obesity who received intensive lifestyle intervention for weight loss had a lower HbA1c, reduced sleep apnoea, reduced diabetes medication requirements, improved mobility and quality of life, fewer hospitalisations, and reduced healthcare costs [9]. With regards to bariatric surgery, a study of 5-year outcomes comparing bariatric surgery to medical therapy revealed that participants with type 2 diabetes who underwent surgery alongside medical therapy were far more likely to achieve the HbA1c < 6% target than those receiving medical therapy alone [32]. In addition, a post-hoc analysis of participants from the Swedish Obese Subjects Study revealed that bariatric surgery was associated with a reduced risk of microvascular complications in patients with diabetes [33]. Bariatric surgery data have also shown that diabetes duration is important in predicting who is likely to achieve diabetes remission [16]. The DiRECT and DIADEM-1 trials also highlighted the importance of intervening soon after the diagnosis of T2DM for diabetes remission with weight loss [10, 11]. Thus, these studies suggest that the presence of obesity should be recognised early on in type 2 diabetes and managed alongside glycaemia, with timely referrals to obesity clinics or for bariatric surgery if appropriate, as significant sustained weight loss can improve glycaemic control, overall health, and lead to remission of diabetes. Recognising this, the local service is now piloting a Diabetes Remission Service in collaboration with primary care, based on the DiRECT study model.

The American Diabetes Association (ADA) guidelines recommend the use of weight lowering anti-diabetes medications in people with T2DM and obesity [34]. In our BMI ≥ 35 kg/m^2^ group, use of weight-lowering medications was limited with less than 1 in 5 participants on a GLP1 agonist and only a quarter on an SGLT2 inhibitor. Data from the Australian National Diabetes Audit (ANDA) showed that in 2019, 27% of people with T2DM were on an SGLT2 inhibitor and 12% were on a GLP1 receptor agonist [35]. Although our use of weight lowering medications in the BMI ≥ 35 kg/m^2^ group was comparable to that of the ANDA, the figures in our specialist clinic should have been higher as the mean BMI of the ANDA population was only 33.5 kg/m^2^ [35]. However, some of these agents like GLP1 agonists and SGLT2 inhibitors are relatively new and are more expensive than the traditional therapies including sulphonylureas and insulin, which treating clinicians may be more familiar with. Unlike the weight-lowering agents, sulphonylureas are associated with weight gain [34]. In our BMI ≥ 35 kg/m^2^ group, sulphonylurea use was appropriately lower than in the BMI < 35 kg/m^2^ group.

This study also showed that new patients to the clinic saw an improvement in HbA1c in the first year, but there was no ongoing further improvement in glycaemia, although the initial benefit seemed to be maintained. We have recently evaluated a locality-based integrated diabetes care service in a neighbouring local government area which showed similar results where even a few years after patients were discharged following specialist diabetes team review, their HbA1c reductions were maintained [19], raising the potential for early discharge of patients from the T2DM clinic after several visits, to create more capacity in the T2DM clinic without compromising patient care. As a result, this would increase capacity in the already strained public T2DM clinic.

This study has some limitations. This is a single centre study of a publicly funded specialist diabetes clinic. However, there are several endocrinologists practising within this clinic. A proportion of people were excluded from the study as their records did not contain a height or weight. There was also a lack of serial weight measurements for the majority of patients which may indicate that staff in the specialist clinic were more focussed on glycaemic control rather than obesity management. Furthermore, some people only attended the clinic once, which made it difficult to assess their progress in regards to weight loss and glycaemic control. The retrospective study design does not account for discussions regarding bariatric surgery or referral to a metabolic clinic which were not documented. A major strength of this study is that all eligible people who attended the clinic were included in the study.

## Conclusions

The results from this study provide a basis for future policy and clinical practice. Many people who attended the public T2DM clinic met the NHMRC criteria for bariatric surgery, but were often not offered a referral to an obesity service or had bariatric surgery discussed as a therapeutic option. This study suggests opportunities for improvement in care of people with T2DM at several levels. Increased referrals from T2DM services to weight management/bariatric services is recommended, along with an increased use of GLP1 agonists and SGLT2 inhibitors where appropriate. The potential for discharge of people who have been treated for over a year in a specialist service has also been highlighted, as has the importance of early recognition of T2DM and management of weight in primary care to provide a chance for diabetes remission, given the long duration of diabetes among the patients seen in a specialist T2DM service. Therefore, the results of this study support the need to systematically consider obesity management in the overall structured management of T2DM.

## Data Availability

The datasets used and/or analysed during the current study are hospital patient data that would not be freely available. For further details of the type of data used and any specific questions, please contact the corresponding author.

## Abbreviations

T2DM: Type 2 Diabetes Mellitus
BMI: Body Mass Index
GLP1: Glucagon-like Peptide 1
SGLT2: Sodium/glucose Co-transporter 2
UKPDS: United Kingdom Prospective Diabetes Study
EASD-ADA: European Association of the Study of Diabetes and American Diabetes Association
NHMRC: National Health and Medical Research Medical Council
BP: Blood Pressure
LDL: Low density Lipoprotein
HDL: High Density Lipoprotein
eGFR: Estimated Glomerular Filtration Rate
ACR: Albumin/Creatinine Ratio
DPP4i: Dipeptidyl-peptidase 4
NDA: National Diabetes Audit
ANDA: Australian National Diabetes Audit
